# EMI reduction method of CISPR 36 pre-compliance testing using affordable rubber-based materials

**DOI:** 10.1016/j.mex.2025.103760

**Published:** 2025-12-10

**Authors:** Arief Rufiyanto, Gamantyo Hendrantoro, Reza Septiawan, Eko Setijadi, Budi Sulistya, Sardjono Trihatmo

**Affiliations:** a Department of Electrical Engineering, Institut Teknologi Sepuluh Nopember, Sukolilo, Surabaya, 60111 Jawa Timur, Indonesia; b Research Center of Electronics, National Agency for Research and Innovation (BRIN), KST Habibie, Serpong, 15314, Banten, Indonesia

**Keywords:** CISPR 36, Electronic module, Electric vehicle, EMI reduction method, Pre-compliance testing, Rubber-based materials, EM Shielding

## Abstract

Electric motor performance is greatly affected by emissions from the automotive drive system (drivetrain), necessitating research to mitigate electromagnetic interference (EMI). This study proposes a set of methods that employs simple and inexpensive rubber-based materials as shielding to reduce EMI in electric vehicle modules and further explores suitable materials to reduce emissions. The effectiveness of three different rubber compositions as shielding EMI, focusing on the frequency ranges regulated in the CISPR 36 standard, is investigated as pre-compliance testing in radial and transversal orientations of the measurement antenna. The study shows that using these methods together, the rubber-based materials under test can reduce EMI emissions by shielding effectiveness (SE) from 37.742 dB to 37.362 dB for single layer and 74.874 dB to 75.479 dB for combination of 2 layers with up to 50 % probability across several frequency ranges, especially the frequencies regulated in the CISPR 36 standard.

A realistic method to provide a reasonably cost-effective solution to reduce EMI, particularly for electric cars in the pre-compliance stage, using simple and inexpensive materials, mainly rubber-based materials,

EMI mitigation method using organic material as an absorber for pre-compliance testing in the frequency range of the CISPR 36 standard,

Method to determine the best combination of materials to reduce the emissions that arise from the electrical module of the DUT.

## Graphical abstract

**Figure:**
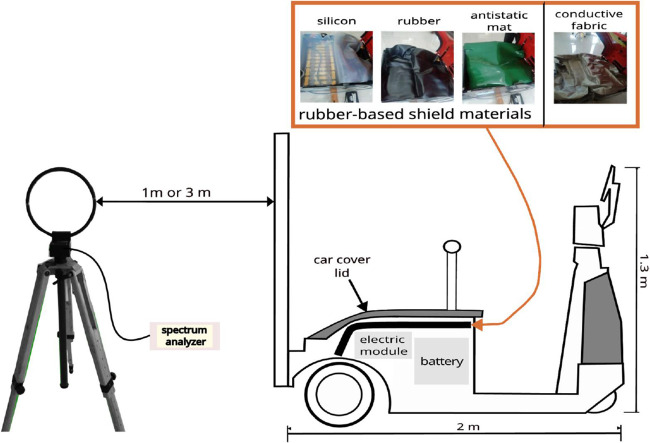

**Table:** Specifications table.

**Subject area**	Engineering
**More specific subject area**	Electromagnetic compatibility
**Name of your method**	EMI reduction method of CISPR 36 pre-compliance testing using affordable rubber-based materials
**Name and reference of original method**	IEC, “Electric and Hybrid Electric Road Vehicles - Radio Disturbance Characteristics - Limits and Methods of Measurement for the Protection of Off-board Receivers Below 30 MHz,” IEC CISPR 36-2020, (2020).
**Resource availability**	None.

## Background

The soaring production of electric vehicles (EVs) has sparked concerns in the transportation industry and among safety advocates. Extensive research has identified various strategies to mitigate EMI in and around EVs, such as shielding, filters, circuit modifications, and spread spectrum techniques (referenced in [1]). This approach concentrates on using the EMI absorber method for low frequencies within the range of 0.15 MHz to 30 MHz, particularly those regulated by the CISPR 36 standard [2].

Several studies related to EMI shielding based on metal-based materials, conductive polymers, and recent developments in organic EMI absorbers have been conducted. Metal coated EMI shielding to reduce exposure of electromagnetic pollution index (EMPI) was compared in [3] to monitor the EMPI components from mobile phones, laptop and microwave using metal filtering and coating method. The three EMPI components observed were magnetic field strength (mA/m), electric field strength (V/m) and radiation, and power density (W/m^2^). There are four shielding materials as metal coating materials have been investigated, namely alumunium foil, silver film SSiSR PS4, LLumarfilm and glass. The results showed that silver film SSiSR PS4 coating has the smallest reduction factor of EMPI in the mobile frequencies 1.4 GHz and 1.8 GHz, while Aluminium foil coating is effective to reduce EMPI sourcing from Laptop processors with 2 GHz speed and from microwave in 2.450 GHz. The shielding effectiveness of very thin and transparent Cu metal mesh with polyethylene terephthalate (PET) was tested with crack templating technique method to create flexible and transparent EMI shielding film in [4] for frequency range of 12 GHz – 18 GHz. The thickness of this shielding material was 450 nm, which has approximately 48 dB shielding effectiveness in 18 GHz with transparency of approximately 85 %. Another metal based shielding, borophene, reference in [5] and achieved a TerraHertz EMI shield effectiveness (SE) of 70 dB and EESt of 4.8 × 105 dB·cm^2^/g (@0.87 THz), which may become an important alternative of shielding material in THz frequency in the future. The SE was analysed with experimental synthesis and simulation to achieve high EMI shielding efficiency in the terahertz. Another material used as shielding material in [6] is a porous sintered Ag layer. This shielding layer has exhibited a high average EMI shielding efficiency of 56.5 dB over a broad frequency range of 0.5–18 GHz, which means EM waves were blocked over 99.99 %. This porous sintered Ag layer was processed with Ag NP dispersions followed by low-temperature sintering and measured with near-field SE mapping method of the porous sintered Ag layer coated PCB antennas. Other metal based shielding material is studied in [7] in form of a metasurface shielding material for WLAN (2.4 and 5.8 GHz) and X-band (8–12 GHz) EMI shielding applications. The shielding is constructed as a double-sided planar metasurface constructed on a Rogers 5870 substrate, which is designed using electromagnetic simulation and fabricated via standard PCB techniques to develop a compact, polarization-independent EMI shielding filter. The transmission response of this of the proposed metasurface based shielding prototype was calculated by measuring TE/TM polarization in an anechoic chamber. The measurement method comprised of two standard gain horn antennas (transmitting and receiving antennas), connected to the Vector Network Analyzer (VNA) using coaxial cables and the prototype is positioned between the antennas. Another high frequency EMI shielding characteristic of a metal based material was studied in [8], which is a nanocomposite of magnetic-ceramic/conductive FeNi3-NiFe2O4-SiO2 NPs and FeNi3-NiFe2O4-SiO2/MWCNT. The fabrication of this shielding material used a one-pot synthesis of magnetic/ceramic nanoparticles followed by surface decoration onto MWCNTs to produce a high-performance EMI shielding material with enhanced conductivity and magnetic loss mechanisms. The concerned frequency was in the range of X (8-12 GHz) and Ku (12-18 GHz) frequency band and is a potential shielding material which blocked 95 % of incident wave. The electromagnetic attenuation characteristics of nanomaterials was calculated based on measurements of the permittivity and permeability parameters which were measured by network vector analyzer. A Literature review of comparison between a metal based shielding material (ferrite and rare earth materials) and organic based materials (biochars and scaffolds) in [9] showed various preparation methods, structure, EMI shielding performance, EMI shielding mechanisms, and application perspectives of these materials. Another alternative for shielding material in the frequency range between 8 GHz and 12 GHz was a combination of polyurethane (PU) foam granules, bio-epoxy composites, and pure vermiculite particles, which has been characterize in [10] using comprehensive characterization techniques, including high-resolution scanning electron microscopy (HR-SEM) and Fourier transform infrared spectroscopy (FTIR), to evaluate the EM shielding properties. This composite fabrication involved mechanical mixing followed by casting and curing. The PU foam combined with bio epoxy materials has potential applications in battery enclosures and cabin soundproofing in electric vehicles. More superior than common pure metal based shielding material, according to [11], is a metal based shielding flexible material by crosslinking polyacrylonitrile (CPAN) nanofiber (NF)/metal nanoparticle (MNP) hybrid membranes. The EMI shielding efficiency is due to the high conductivity of MNPs and favorable porous structure in the rectangular shaped piece of hybrid NF membrane and was measured by the rectangular waveguide method using a 2-port network in the frequency range of 8–26.5 GHz (X-band (8–12.4 GHz), Ku-band (12–18 GHz), and K-band (18–26.5 GHz)). A comprehensive review of EM shielding is discussed in [12] from various shielding materials based on carbon, graphene, and activated carbon, a special attention was given to carbon polymer hybrid nanocomposites since their flexibility and high EM shielding effectiveness in frequency range of 8.2 GHz-12.4 GHz.

Several researchers have adopted a conventional approach, developing rubber-based composites to create flexible materials by incorporating magnetic or ferrite particles. Researchers in [13] has evaluated noise-suppressing sheets made of flexible polymer-magnetic particle composites for reducing automotive EMI. Using CST simulation and measurement, the study showed their effectiveness in the 30 MHz–1 GHz range, offering a flexible solution to improve vehicle EMC. Reference [14] proposes a framework for evaluating magnetic absorbing materials that considers both reflection loss (RL) and surface wave attenuation (SWA). This approach provides a detailed understanding of material performance, considering characteristics in both the far-field and near-field environments. It is effective for optimizing microwave absorption within the 0.3–18 GHz range. The research presented in study [15] investigated natural rubber-based materials for electromagnetic wave absorption. The study demonstrated that incorporating conductive fillers such as carbon black and Fe₃O₄ effectively blocks electromagnetic waves across a broad frequency range. One study presented in [16] examined flexible rubber sheets as EMI reducers. The structure of a polymer matrix embedded with magnetic particles demonstrated flexibility and ease of integration into electronic systems and was evaluated across a frequency range of 30 MHz to several GHz. Paper [17] details the development of a wide-band electromagnetic wave absorber utilizing rubber-ferrite composite materials. The material was fabricated through conventional ceramic processing and exhibits flexibility, lightweight properties, and effective attenuation across the 2–18 GHz frequency range. The study in [18] proposes an absorber composed of neoprene rubber layers with a 3D-printed polylactic acid (PLA) layer filled with charcoal powder sandwiched in between, designed for EMI/EMC applications. The explored performance suggests this absorber can serve as an environmentally friendly alternative for EMI shielding. A study detailing the development of radar absorbers utilizing rubber latex and coconut shells as fillers is presented in [19]. The microwave properties of the absorbers were characterized using a microwave non-destructive testing (MNDT) technique. This study also investigated the influence of bandwidth, carbon content, and absorber thickness on performance.

In the emerging eco-friendly solutions, some researchers have presented the newest and most enviromentally conscious direction in EMI shielding. Researcher in [20] developed a nacre-inspired starch-based bioplastic with high mechanical strength and effective EMI shielding. Mimicking nacre’s structure, it offers flexibility and durability, serving as a sustainable alternative for EMI shielding in electronic devices. A bio-inspired, multifunctional EMI shielding coating created in [21] using layer-by-layer construction. The material has excellent shielding performance and durability under extreme conditions such as radiation, vacuum, and temperature extremes, making it ideal for aerospace and satellite applications that require reliable EMI protection. Bio-composites with efficient heat dissipation and excellent EMI shielding developed in [22] by integrating bamboo fibers with MWCNTs in a dual network structure. This design combines the mechanical strength and sustainability of bamboo with the high conductivity of MWCNTs, offering a lightweight, eco-friendly solution for EMI shielding in electronic devices that require both thermal management and electromagnetic protection. Another study [23] examined how thickness and conductivity affect the EMI shielding effectiveness of bio-composite materials. The study showed that increasing material thickness and electrical conductivity improves EMI performance, especially in the X-band (8.2–12.4 GHz). These findings support the development of eco-friendly, sustainable EMI shields for use in electronic devices and green technologies. Researchers in [24] developed epiphyte-inspired multifunctional biocomposites for EMI shielding. The materials combine natural fibers with conductive fillers to enhance mechanical strength and EMI performance. The biocomposites offer a lightweight, flexible, and sustainable solution, showing effective EMI shielding in the X-band (8.2–12.4 GHz), suitable for green electronics and biodegradable shielding. The EMSE of biochar-based materials as sustainable EMI shielding solutions are evaluated and tested in [25]. Tested across X-band (8.2–12.4 GHz), S-band, and Ku-band, the materials show promise for broadband EMI suppression.

To address the challenge of electromagnetic interference (EMI) shielding, researchers are increasingly investigating the potential of metamaterial and metasurface structures as advanced technical solutions. Study explored the application of metamaterial structures to enhance the shielding of Bluetooth antennas in electric vehicles [26]. Researchers engineered metamaterials to manipulate electromagnetic waves, thereby improving signal integrity and reducing interference at 2.4 GHz. Reference [27] presents the design of a metasurface absorber capable of operating across three bands, targeting 5G/6 G millimeter-wave applications. Using multi-resonance design and parameter optimization, researchers created a thin, compact structure that provides broad absorption and is not sensitive to polarization. Researchers [28] investigated RF absorbers to reduce radiated emissions from electric vehicles. Using a combination of measurements and simulations performed with CST software, the study focused on the 30 MHz–1 GHz frequency range.

EMI filtering involves embedding or leveraging filters to mitigate outlier noise [29]. Nonlinear filtering methods are used to reduce interference in communication systems and sensors [30]. Filters are crucial in automotive systems to reduce AM frequency band interference, specifically from 0.15 kHz to 280 kHz and from 0.53 MHz to 1.7 MHz [31]. The primary goal of EMI filtering is to mitigate interference in the low-frequency range.

Electrical circuit modifications in electronic products can help mitigate EMI. Studies have shown that excessive hub loads on electric machines can lead to issues and require measures to minimize them [32]. Studies on EVs equipped with dielectric panels using different chassis materials, shielding, altering current loop areas, and incorporating battery shields [33]. EMI from AC motor drive systems can also be mitigated by incorporating Y capacitors and ferrite [34]. Regulations on drivetrain systems prevent interference with the surrounding electromagnetic atmosphere [35]. Noise reduction methods, such as enhanced capacitor arrangement and improved CM choke design, are effective [36]. Layout and EMI electrical circuit enhancement can mitigate EMI in the low- and medium-frequency ranges [37].

The spread spectrum method aims to minimize electromagnetic interference by modifying the spread of the spectrum signals. Research has shown that the spread spectrum clock generation (SSCG) used in a radiation geometry model and NEC-4 software reduces irradiated emissions from devices using digital clock signals [38]. Real-time outlier noise reduction can be achieved through excess band observation of outlier noise [29]. EMF concerns in EVs can be addressed through spread spectrum techniques in DC/DC converters, particularly those using pulse-width modulation [39] and resonant converters [40]. This method is primarily used in the low-frequency range.

This paper describes the EMI pre-compliance testing method of the CISPR 36 standard [2] for electric vehicles, with the goal of mitigating EMI using an organic-based rubber material as an EM shield/absorber for both the transversal and radial orientations of the measurement antenna. As mentioned earlier, some EMI mitigation methods aim to reduce EMI emissions in the low-frequency range by using the EMI filter method, the EMI electrical circuit method, and the EMI spread spectrum method. In contrast, extensive research on EMI mitigation methods using EMI shielding or absorbers has primarily focused on frequency ranges above 30 MHz. There is limited research on EMI mitigation methods using EMI absorbers in the frequency range specified by CISPR 36.

This work has novel contributions as follows:

• A realistic method has been proposed in this paper to provide a new reasonably cost-effective solution to reduce EMI, particularly for electric car goods in the pre-compliance stage, using very simple and inexpensive materials, mainly rubber-based materials.
• EMI mitigation method in this paper has novelty in using organic material as an absorber for pre-compliance testing, especially in the frequency range of the CISPR 36 standard.
• A novel method to determine the best combination of materials to reduce the emissions that arise from the electrical module of the DUT.

This pre-compliance testing method can be used to mitigate the radiated emissions from commercial electric vehicle electronic modules, since there are still some potential emissions that may not be fully addressed by the manufacturer and may occur after the product is put on the market.

The proposed method in its entirety is composed of four components having their respective validation tests, i.e.,

- Method to characterize materials
- Method to measure volume resistivity
- Method to measure shielding effectiveness
- Method to measure EMI pre-compliance by applying materials on electronic modules

The proposed approaches are described in detail below.

## Method to characterize materials

A better understanding of potential methods to reduce EMI using rubber-based materials can be achieved by investigating fundamental references. Previous extensive fundamental research has been conducted on EMI mitigation techniques in some references. A near-field scanning method for minimizing the radiated emissions of products was explained in [41], which validated this by performing far-field measurements within a semi-anechoic chamber. This method is more economical than the conventional approaches. Radiated emission issues at the vehicle level have been identified in [42] by highlighting critical safety measures and efficient data collection methods for non-compliant vehicles during testing. Retesting at the vehicle level is consequently circumvented, allowing mitigation strategies to be implemented at the supplier level. In addition to the standard MIL-STD-461 for emission testing, paper [43] introduced the Sniff Test (ST). The key aspect of emissions reduction in automotive contexts caused by the AM band (0.15 kHz to 280 kHz) and the medium-wave AM band (0.53 MHz to 1.7 MHz) is discussed in [31]. In addition, [44] proposed a method to minimize electromagnetic interference by optimizing the spacing between the windings and shielding. In relation to the development of rubber and its derivatives as an alternative shielding material, many studies have been conducted on this matter. Rubber and carbon black filler materials have been measured for reflectivity and transmissive parameters to extend the shape of the pyramid absorber in the absorbers’s manufacturer [45]. In addition, the use of natural rubber mixed with activated carbon from bamboo for absorbent manufacturers has also been studied in [46]. Five formulas were used, with amounts varying from 0 parts per hundred of rubber (phr) to 60 phr. From the results of the reflectivity measurements, it is concluded that the number of mixtures shows better results, and when compared to the commercial absorbers, the results are better. In [47], a flat microwave absorber working at a frequency of 1.8 GHz was developed by the impregnation of activated carbon and polyurethane. The activated carbon is prepared by the conversion of the rubber wood sawdust through a chemical activation process using a ZnCl_2_ solution as the precursor agent. Based on the BET analysis, the surface area of the powder for the impregnation ratio 1.5:1 reported the highest value of 1301 m²g⁻¹. This formulation was selected for making microwave absorbers, and it was reported that the reflection loss was recorded at 10 dB at 1.8 GHz. In [48], it was shown that controlling the composition of MnZn in ferrite-rubber composites affects the reflection loss (about −40 dB in the frequency range of 0.8 GHz to 12 GHz). In [49], epoxy-modified urethane rubber was mixed with carbon particles at a frequency of 60 GHz.

Some researchers have, moreover, employed simulation techniques using shielding to accelerate EMI mitigation, including [50], which details the improvement of shielding effectiveness using CST simulation software. The study in [51] focused on modeling EMI filters with integrated shielding between components. Similarly, [52] investigated the design and development of flexible, lightweight carbon nanofiber for EMI shielding applications and analyzed their effectiveness through CST simulations. Finally, [53] examined the behavior of electromagnetic shielding from enclosures with apertures using CST to replicate shielding effectiveness.

**Fig. 1:**
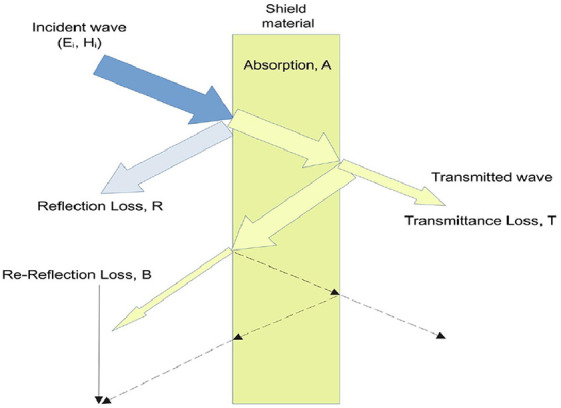
Wave interaction on the surface of a shielding material.

The shielding theory is derived from Maxwell’s equations. Schelkunoff derived the shielding effectiveness of an infinitely spread thin surface from shielding theory in 1943 [54], and a further study of the shielding was done in [55]. Fig. 1 shows the wave interaction on the surface of a shielding material.

The wave interaction on the surface of a shielding material essentially varies with the EMI shielding effectiveness of the material itself. EMI shielding effectiveness (EMI SE) is a classification of a material’s capacity to shield by reflecting or absorbing and possibly emitting just a tiny portion of the energy. In dB units, the EMI SE is defined as the ratio of the EM power before the shielding effect to the EM power after shielding. The shielding effectiveness and efficiency were calculated from the measured scattering parameters as follows [[56], [57], [58]], and [59]. The shielding effectiveness is classified into the shielding effectiveness of reflection (SER) and the shielding effectiveness of absorption (SEA).

(1) $$SER=\;-10\;x\;log_{10}\left(1-R_{efl}\right)$$

(2) $$SEA=\;-10\;x\;log_{10}\left(\frac{T}{R_{efl}}\right)$$

Reflectance, *R_efl_*, an absolute value of the reflection coefficient, *S*_11_, is associated with SER. Transmittance, T, an absolute value of the transmittance coefficient, *S*_21_, is connected to SEA.

(3) $$R_{efl}={\left|S_{11}\right|}^{2}$$

(4) $$T={\left|S_{21}\right|}^{2}$$

(5) $$A_{abs}=1-\left(R_{efl}+T\right)$$

The transmitted signal is reflected with a reflection loss, *R_L_* is a loss due to the first reflection at both surfaces of the boundary. and is absorbed with an absorption loss, *A_L_* is an absorption loss during a single transient through the boundary of the shielding material as follows:

(6) $$R_{L}=\;-20\;x\;log_{10}\left|S_{11}\right|$$

(7) $$A_{L}=\;-20\;x\;log_{10}\left|S_{21}\right|$$

The total loss is the sum of absorption loss, *A_L_*, and reflection loss, *R_L_*.

This section will briefly describe the materials used for shielding in electronic modules in electric vehicles. Table 1 describes material specifications and sources of these materials. The study materials were purchased directly from the market. Their specifications came from market websites. These materials needed to be characterized to determine their properties, such as permittivity, conductivity, resistivity and shielding effectiveness, for use in the study. These materials were characterized by measuring volume resistivity, electrical properties and performing Fourier Transform Infrared (FTIR) spectroscopy. Shielding effectiveness was tested within a spesific chamber.

**Table 1:** Specification of material types.

Type of material	Description
Silicon (S) [60]	Reinforce: insert 1 ply cloth. Tensile Strength: 7 MPa to 12 MPa. Hardness: 40, 50, 60, 70, 80 ± 5 shore A Elongation: 350 % to 720 %. Density: 1*.*25 gcm^−3^ to 1*.*50 gcm^−3^. Tear Strength: 26 Nmm to 42 Nmm. Working temperature: −60°C ± 260°C (Max moment 300°C).
Rubber (R) [61]	Working temperature: −35°C to 85°C.
Antistatic Mat (A) [62]	Voltage range: 5 kV to 50 kV (depending on color). First layer thickness: 0.5 mm (layer statis dissipative) Second Layer thickness: 1.5 mm (conductive layer).
Conductive Fabric (F) [63]	Fabric thickness: 0.07 mm. Surface resistance: below 0.05 Ω Frequency Range: 30 MHz to 35 GHz Damping: 85 dB to 65 dB Material (Copper, Nickel, Polyester).

The main materials’ resistivity (ρ in Ω.m) and conductivity (σ in S/m) are known from [64]. Then, the shielding effectiveness of a material depends on resistivity (ρ), material thickness (d), and the frequency of the incident wave (f_i_). When an incident wave of frequency, *f,* attains the surface of a material with thickness d, then the shielding effectiveness of the material follows the equation given in [65], and Eqs. (6) and (7) can be rewritten as:

(8) $$SE_{total}=\;SER+\text{SEA}+B\;\left(\text{dB}\right)$$

Where: *R_L_* is the reflection loss as in Eq. (6) and related to resistivity according to the following equation:

(9) $$R_{L}=\;-20\;x\;log_{10}\frac{{\left(\alpha +1\right)}^{2}}{4\;x\;\left|\alpha \right|\;}$$

*A_L_* is the Absorption Loss as in Eq. (7) and related to resistivity and thickness d as follows:

(10) $$A_{L}=\;8.686\;x\;\alpha \;x\;d\;\left(\text{dB}\right)$$

The constant, *α*:

(11) $$\alpha =\;{\left[\frac{\mu \sigma \omega }{2}\right]}^{\frac{1}{2}}=\;\frac{1}{\delta }$$

where thickness of barrier (meters) is d, distance from source to barrier (meters) is r, *ω* = 2 × *π* × f, absolute permeability of barrier (H/m) is *µ* (12*.*56 × 10^7^), absolute conductivity of barrier (℧/m) is *σ*, and *λ* = $\frac{c}{f}$ =

$\frac{3\times 10^{8}}{f}$ (meters). B is re-reflective coefficient when the thickness of the barrier is thin (less than 2*πδ*).

(12) $$B=20\;x\;log_{10}\left[1-{\left(\frac{k-1}{k+1}\right)}^{2}e^{\left(-2d/\delta \right)}\right]$$

where:

(13) $$k=\;\frac{Z_{wave}}{Z_{barrier}}$$

The equation for the impedance of the barrier (consistent with shielding effectiveness theory) is [45]:

(14) $$Z_{\text{barrier}\;}=\frac{1+j}{\sigma \delta }$$

For high impedance source:

(15) $$Z_{wave}\approx -j\;\frac{377\;x\;\lambda \;}{2\pi r},\;\left(r<\frac{\;\lambda \;}{2\pi }\right)$$

For low impedance source:

(16) $$Z_{wave}\approx j377\;\left(\frac{2\pi r\;}{\lambda }\right),\;\left(r<\frac{\;\lambda \;}{2\pi }\right)$$

For all sources:

(17) $$Z_{wave}\approx j377,\;\left(r\geq \frac{\;\lambda \;}{2\pi }\right)$$

The above expression explains the relationship between the shielding effectiveness and the conductivity of an absorber material, which relates to the conductivity, thickness of the material, frequency, and distance.

Table 3 shows the results of calculating the shielding effectiveness in dB of the material using the resistivity and conductivity [64] and the Eqs. (8) to (17) at a frequency of 10 MHz. The conductive fabric is made mostly of copper and nickel. This gives it very low resistivity. This synthetic fabric acts as a conductive material and absorbs low-frequency range emissions. The waves coming in on this conductive material move straight across the fabric. In contrast, silicon has a resistivity value that is 10^9^ times higher than that of conductive fabric. The rubber and PET materials have resistivity values of 10^21^ and 10^28^ times that of the conductive fabric. The conductive fabric has very low resistivity. So, S_11_ of this conductive fabric should also be low since the incoming waves get absorbed by the copper and nickel particles. In contrast, rubber also has low S_11,_ despite it has high resistivity, since the incoming waves cannot move freely on the surface.

## Validation of materials characterization method

The absorption coefficients for rubber, antistatic mat, and silicon materials varied by less than 21.94 %. The average difference between the measurement and the calculation was 3.67 dB.

Table 4 display the deviation value of the shielding effectiveness from the calculation Eq. 9 for R_L_ and Eq. 10 for A_L_ and measurement Eq. 3 for R_efl_ and Eq. 4 for T at a frequency of 10 MHz.

The source of deviation is the referred [64] absolute conductivity of the barrier (1/Ω m) of materials (copper, silicon, nickel, rubber, and PET). Three main factors affect this conductivity: cross-sectional area, length of the conductor, and temperature. The three main factors may vary in our measurement; for instance, in [64] the temperature was 20° C.

The measured absorption coefficient varies from the theoritical values: 3.74 dB for silicon, 3.42 dB for rubber, and 3.84 dB for antistatic mat, see Table 4. Reference [64] observed that rubber has a resistivity of 10^13^. This differs from the resistivity measurements, which show it around 10^10^ (see Table 2). The calculated (T) and the observed measured effectiveness (AL) come from accurate values provided by absorbed materials manufacturers for the input variables of (9), (10).

**Table 2:** Resistivity and conductivity of material [64].

Material	*ρ* (Ω*.m*)	*σ* (*S/m*)
Copper	1*.*68 × 10^−8^	5*.*96 × 10^7^
Nickel	6*.*99 × 10^−8^	1*.*43 × 10^7^
Silicon	6*.*40 × 10^2^	1*.*56 × 10^−3^
Rubber	10^13^	10^−14^
PET	10 × 10^20^	10^−21^

**Table 3:** Calculation shielding effectiveness material at 10 MHz (in dB).

Material	*R_L_*	*A_L_*	B	*SE_total_*
Silicon (S)	2*.*52	0*.*013	−0*.*23 × 10^3^	2*.*53
Rubber (R)	−1*.*20	0*.*36	0*.*10	−1*.*10
Antistatic Mat (A)	−1*.*20	0*.*75	0*.*20	−0*.*99
Conductive Fabric (F)	1*.*73 × 10^2^	2*.*71 × 10^5^	0*.*30	2*.*72 × 10^5^

**Table 4:** Deviation of shielding effectiveness between measurement and calculation.

Material	*R_L_*	*A_L_*	*Refl*	T	*Refl*-*R_L_*	T-*AL*
Silicon (S)	2*.*73	1*.*67	1*.*37	−3*.*75	−1*.*36	3*.*74
Rubber (R)	−1*.*19	4*.*47 × 10^−5^	1*.*35	−3*.*42	0*.*16	3*.*42
Antistatic mat (A)	−1*.*19	9*.*45 × 10^−5^	1*.*37	−3*.*84	0*.*18	3*.*84
Conductive Fabric (F)	1*.*75 × 10^2^	3*.*40 × 10^5^	1*.*41	−3*.*86	−1*.*60 × 10^2^	−3*.*40 × 10^5^

## Method of the volume measurement resistivity

In the volume measurement resistivity, the parameters measured the insulation resistance, *R_ISO_*, and the dielectric absorption ratio, *R_ad_*. These parameters are calculated as [66]:

(18) $$R_{ISO}=\;\frac{U_{ISO}}{I_{M}}$$

where R_ISO_ determined from the measuring voltage, U_ISO_ and the test current, I_M_.

(19) $$R_{ad}=\;\frac{R_{ISO}\left(60s\right)}{R_{ISO}\left(30s\right)}$$

where *R_ISO_(30* *s)* and *R_ISO_(60* *s)* are the insulation resistance test measurements at 30 and 60 seconds respectively (in Ω).

**Fig. 2:**
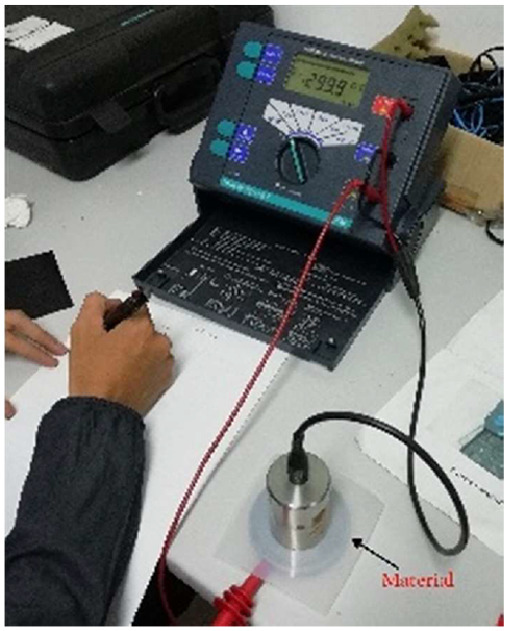
Photograph of resistivity volume measurement setup.

Fig. 2 shows a photograph of volume resistivity measurement.

The procedures used to measure volume resistivity are as follows:

1. Set up the measurement tool and arrange the measuring setup as indicated in Fig. 2 with the materials to be tested.
2. Adjust the tool voltage on the central switch. This way will provide maximum insulation. Then, the voltage and insulation resistance (R_ISO_) values can get.
3. Based on the findings of the third step, use the tool's R_ad_ field function to calculate the R_ad_ values. In this measurement, the testing device is used. Insulation resistance is measured at 30 seconds and again at 60 seconds.
4. Take measurements three times for each material and at different positions.
5. Apply steps 1–4 for different materials.

## Validation of volume measurement resistivity method

Silicon has the highest resistivity, reaching up to tera-ohm. It can also withstand a voltage of up to 1000 V, as shown in Table 5. While, according to [64], the resistivity used to measure silicon is 10^2^ (see Table 5). The dielectric adsorption ratio, R_ad_, is higher silicon than for antistatic mat materials. The average silicon value is 1.037. In comparison, rubber and antistatic mats have values of 1.026 and 0.990, respectively.

**Table 5:** Measurement of the resistivity of materials.

No	Material	Voltage (V)	R_ISO_ (Ω.m)	Rad
1	Silicon (S)	1000	1.697 T	1.037
2	Rubber (R)	500	11.800 G	1.026
3	Antistatic Mat (A)	500	24.897 M	0.990

## Electrical properties

The parameters of the material used in the study, such as permittivity, conductivity, and thickness, measured using a Hioki 3532-50 impedance analyzer and conducted in a controlled environment. The measurements were done at 61% relative humidity and 23.10°C. The electrical properties measured at 1.195 MHz, which is within the CISPR 36 range of 0.15–4 MHz. These results are shown in Table 6.

**Table 6:** Electrical Properties at 1.195 MHz.

Material	Permittivity, *ε_r_****(F/m)***	Conductivity, *σ (S)*	Thickness (mm)
Conductive Fabric (F)	6917.421	1.5369 × 10^-2^	0.088
Rubber (R)	4.553	2.409 × 10^-6^	0.994
Antistatic Mat (A)	13.459	9.277 × 10^-6^	1.824
Silicon (S)	3.153	6.244 × 10^-6^	0.948
Silicon (S)/Rubber (R)	4.953	1.938 × 10^-6^	1.942
Silicon (S)/Antistatic (A)	9.975	1.983 × 10^-6^	2.772
Silicon(S)/ Conductive Fabric (F)	4.306	7.417 × 10^-7^	1.036
Rubber (R)/Antistatic (A)	14.295	4.276 × 10^-6^	2.818
Rubber (R)/ Conductive Fabric (F)	6.786	3.343 × 10^-6^	1.082
Antistatic (A)/ Conductive Fabric (F)	15.462	6.189 × 10^-6^	1.912

EMI shielding is largely determined by three factors: electrical conductivity, permittivity, and thickness. These properties influence how electromagnetic waves interact with the shielding medium. Electrical conductivity plays a dominant role, particularly in reflection-based shielding. High conductivity materials can reflect a significant portion of incident electromagnetic waves. This is especially at higher frequencies. Conductivity is key when designing shields that prevent electromagnetic waves from penetrating sensitive components [23,67]. Permittivity affects how a material stores and dissipates electromagnetic energy. Materials with higher permittivity improve absorption-based shielding by allowing more of the electromagnetic wave to enter and be attenuated within the material. This is particularly important in multilayer or hybrid shielding systems. In these system, dielectric layers are used to trap and dissipate energy through multiple reflections and absorptions [67].

Thickness also contributes significantly to shielding performance, especially absorption. Thicker materials provide better attenuation of electromagnetic waves, as they offer a longer path for energy dissipation. But, the relationship is not linear; beyond a certain point, increasing thickness yields diminishing returns. Also, the concept of skin depth at which the electromagnetic wave’s intensity drops significantly—becomes relevant. For high-frequency signals, thin conductive layers can work well if the material has high conductivity [23]. In practical applications, achieving the best EMI shielding needs a good combination of these three factors.

## Fourier transform infrared spectroscopy

**Fig. 3:**
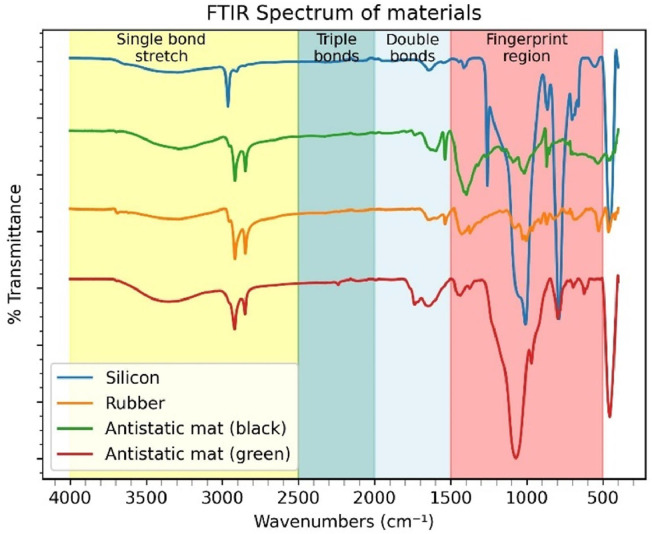
Fourier transform infrared spectroscopy (FTIR) spectrum of material used in the study [68].

Fig. 3 shows the results from the Fourier Transform Infrared Spectroscopy (FTIR) spectrum of the material used in the study. Fourier transform infrared spectroscopy (FTIR) spectrum of material: silicon, rubber, antistatic mat (black), and antistatic mat (green).

In general, the absorption peak of silicon at 2960 cm^-1^ is assigned to the stretching vibration of -CH_3_ . The absorption peaks at 1260 cm^-1^ and 864 cm^-1^ correspond to the bending vibration and rocking vibration of Si-CH_3_ . The peaks at 1080 cm^-1^ and 1010 cm^-1^ show the stretching vibration of Si-O-Si bonds. These bonds are part of the backbone of silicone rubbers. The absorption peak at 793 cm^-1^ comes from to the coupling of stretching vibration of Si-C and rocking vibration of -CH3 [69].

Rubber ashows key absorption peaks around 2950 cm⁻¹ and 2850 cm⁻¹. These peaks correspond to aliphatic C–H stretching bands (–CH₂– and –CH₃). 1650 cm⁻¹ C=C stretching (alkene double bond). 1450–1375 cm⁻¹ C–H bending (–CH₂– and –CH₃). 1000–830 cm⁻¹ shows typical out-of-plane =CH (cis/trans) vibration.It also indicates C–C stretching in polyisoprene chains [70].

The main absorption peaks of the antistatic mat (black) are observed at around: 2950 cm⁻¹ and 2850 cm⁻¹ aliphatic C–H stretching bands (–CH₂– and –CH₃). 1650 cm⁻¹ C=C stretching (alkene double bond). 1450–1375 cm⁻¹ C–H bending (–CH₂– and –CH₃). 1000–830 cm⁻¹ typical out-of-plane =CH (cis/trans) vibration and C–C stretching regions of polyisoprene chains [71].

The spectrum for the antistatic mat (green) is like the black one. However, the C-Cl bands intensities around 814 cm⁻¹ are visible [72].

Silicone rubber stands out among rubber sheets because it has a crystalline structure. The crystalline content boost the material's shielding efficiency, in line with the reference [73]. This materials was chosen for its proven shielding effectiveness, showing values between 20 and 50 dB [74,75]. Natural rubber was chosen for its good shielding ability. Its performance can be enhanced with appropriate fillers [76].

## Method of shielding material effectiveness measurement

**Fig. 4:**
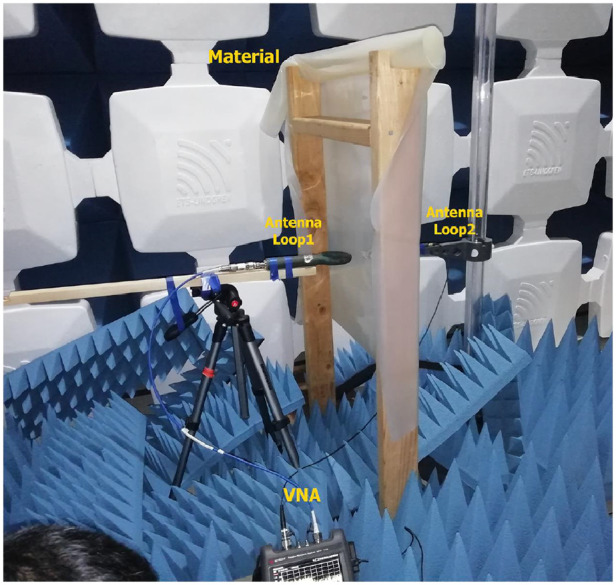
Photograph of the apparatus for measuring the shielding efficacy of the material.

In the material characterization, the measurement method is shown in Fig. 4. Fig. 4 is a setup to measure the SE of the material that will be used with reference to [77] by modifying the distance between the material and the loop antenna is adjusted to 0*.*05 m.

To measure how well the material shields the electronic module, a specific method is used. The tools used included two-loop antennas. One had a frequency range of 20 MHz to 230 MHz. The other ranged from 0.15 MHz to 50 MHz. A Vector Network Analyzer (VNA) and a holder for the material used to be measured.

The measurement steps are as follows:

1. The space between two loop antennas is 0.1 m, and the room's floor height is 0.7 m. Every antenna is linked to the VNA (see Fig. 4). The initial measurement was taken without any materials.
2. Place the material on the holder. Set the distance between the material and the ends of the loop 1 and loop 2 antennas to 0.05 m. Place an absorber around the measuring area to reduce reflections.
3. Material properties are measured at frequencies ranging from 0.15 to 30 MHz.
4. Follow steps 1–3 for each material to be utilized in the research. Besides measurements, conductive fabric is measured. The findings can be served as a reference or help compare with other materials.

## Validation of shielding material effectiveness measurement

**Fig. 5:**
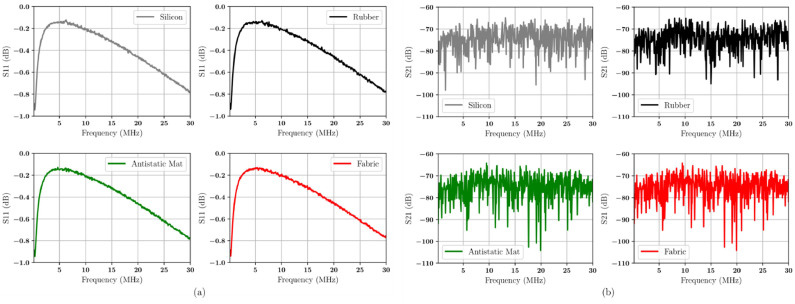
The characteristics of the S-Parameter (a) S11 and (b) S21 in the frequency range of 0.15 MHz to 30 MHz.

The measurement and characterization of the material are shown in Fig. 5. Fig. 5(a) shows the S-Parameter (S_11_) at a frequency range of 0.15 MHz to 30 MHz for silicon, rubber, antistatic mat, and conductive fabric. Fig. 5(b) displays the the S-Parameter (S_21_) characteristics for silicon, rubber, antistatic mat, and conductive fabric. This data covers a frequency range of 0.15 MHz to 30 MHz.

Fig. 5(a) shows S_11_ ranging from −0.03 dB to 0.03 dB. The shielding effectiveness measurements from 0.15 MHz to 30 MHz show that the material transmittance varies from −20 dB to 35 dB (see Fig. 5(b)).

## Method of EMI pre-compliance measurement with single or combined materials

**Fig. 6:**
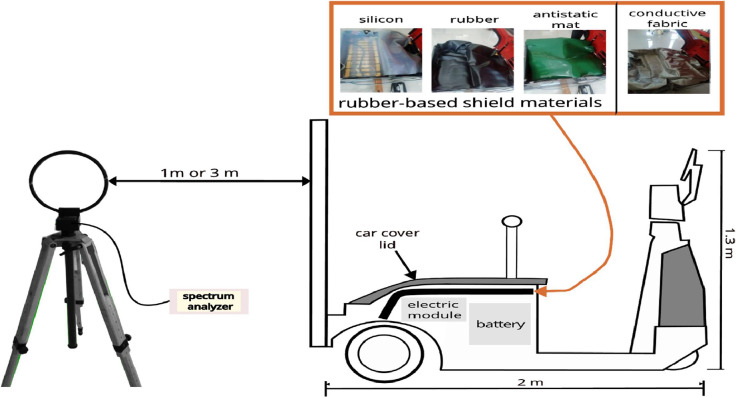
An illustration of the DUT used in the study.

An electric vehicle designed for towing and hauling was used as the device under test (DUT) in this investigation. Fig. 6 illustrates the DUT measurement used in the study. We took measurements at 1 m and 3 m from the DUT in accordance with the CISPR 36 standard [2].

The figure depicts a cutaway view of the measurement setup. All modules, specifically the EM (electrical module) and the battery in the DUT, are covered with rubber-based materials and conductive rubber. This is indicated by the black line in the figure. Finally, they covered the DUT’s outer cover (lid). We make every effort to fully cover the DUT’s electronic and battery components with material. The study uses rubber-based materials. These include silicon, rubber, antistatic mat, and conductive fabric as reference. The goal of covering these electronic modules is to cut down on the emissions they radiated or emitted.

The measuring tools used include the following:

1) Low-cost real-time spectrum analyzer (Signal Hound BB60C) with a frequency range of 9 kHz to 6 GHz
2) Active 12” Loop Antenna SAS-563B with a frequency range of 1 kHz to 30 MHz.
3) Tachometer.
4) Tripod.
5) Measuring tape.
6) Notebook with Spike™ software.
7) Cables and accessories.

Under this method, DUT is measured under a variety of situations or operating modes. The operational modes are the following:

1) OFF Testing Mode: Set the mode of the object under test to the OFF condition. Here, "off mode" refers to disconnecting the battery from the test object. Measurements in this mode are aimed at identifying the local EM environment’s ambient conditions.
2) ON Testing Mode: Perform testing with the condition of the test object in ON mode. The test object switch is still OFF position. However, the test object is now attached or connected to the battery, which is ON. Measurements in this mode help identify radiated emissions from EV batteries and other electric sources.
3) STANDBY Testing Mode: Perform testing with the condition of the test object in STANDBY mode. In this mode, the test object connects to the battery. The test object switches then turns the key to the ON position. This step finds the in situ radiated emissions from electric vehicle batteries and other electric modules when they are in the ON position. Therefore, it is possible to distinguish the radiated emissions that occur in the ON and STANDBY modes. This may show emissions from an active relay.
4) RUN Testing Mode: Test the object while it has in RUN mode. The motor should operate at 200 rpm, as measured by a tachometer. This mode means that the test object is in a static moving condition with the rotation speed set to 200 rpm. This step checks the emissions from the electric car when the motor runs at 200 rpm.

The following are the steps of the proposed test method:

1) Place the measuring antenna at the measuring points. These are 1 m or 3 m from the front, right, rear, and left sides of the test object. In this case, the rear emits the most because the engine is at the back or because of the electrical module's location.
2) Use a loop antenna with radial and transverse orientation. Set the antenna height of 1.3 m. Measure with conditions or mode OFF without covering the DUT electronic modules at a distance of 1 m.
3) Repeat step 2. This time, add one layer of one of a current material type. I can be choosed silicon (S), rubber (R), antistatic mat (A), and conductive fabric (F).
4) Repeat step 3. This time, use a mixture or combination of the types of material instead of just one. Combine types like silicon with rubber (SR), silicon with antistatic mat (SA), silicon with conductive fabric (SF), rubber with antistatic mat (RA), rubber with conductive fabric (RF), and antistatic mat with conductive fabric (AF).
5) After measurement at 1 m is completed, continue with a measurement at 3 m. Use the same methods as steps 2 to 4.
6) Finally, after completing all measurements have been done, calculate the measurement uncertainties of the antenna, cable, and receiver. For calculation use the "Guide to Expressing Uncertainties in Measurements" [78].

**Fig. 7:**
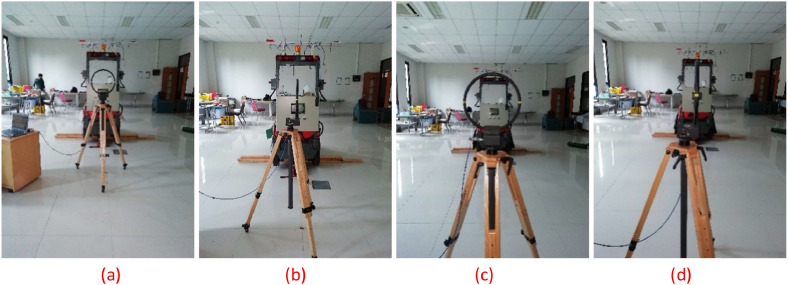
Measurement configurations at distances of 1 m and 3 m with radial and transverse antenna orientations.

Fig. 7 shows four measurement setups: (a) at a distance of 1 meter with a radial antenna orientation, (b) 1 meter with a transverse orientation, (c) 3 meters with a radial setup, and (d) 3 meters with a transverse setup.

Measurements were conducted in normal conditions. Temperatures ranged from 24.7°C to 25°C and relative humidity was between 70 % and 80 %. In this study, all measurements were performed in situ, outside the chamber. The room measured 11.93 m long, 8.51 m wide, and about 3.49 m high.

**Fig. 8:**
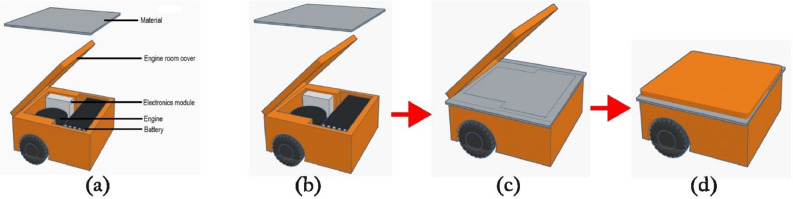
The way material is layered on electronic module of EV.

A single-layer material was placed over the electronic module of the electric vehicle, see Fig. 8(b) and Fig. 8(c), and then the vehicle lid was closed to measure emission attenuation, see Fig. 8(d). The measurement method followed the scenario described in the previous subsection. Following the single-layer test, a two-layer material combination was subsequently evaluated.

Table 7 shows how the material layers were arranged during measurement to shield the EV’s electronic parts.

**Table 7:** Composition of layer when it applies on electronic component of EV.

## Validation of EMI pre-compliance measurement with single or combined materials

**Fig. 9:**
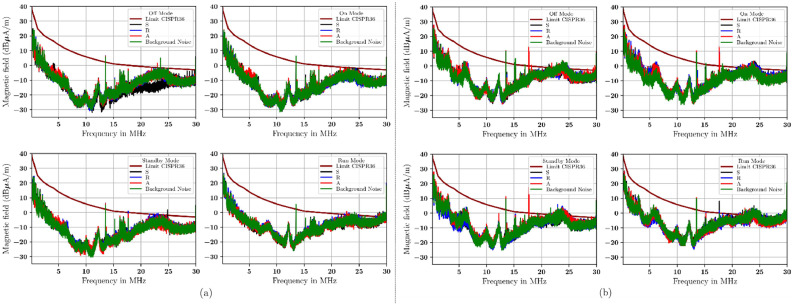
Measurement with (a) radial orientation, (b) transversal orientation at a distance of 1 m using a single-layer material.

Fig. 9 to Fig. 12 show the electromagnetic emission from electric vehicles as DUT. Different modes of operations of the DUT are important. This is a key parametr limited by CISPR 36. The limit of CISPR 36 [2] is given by a brown continuous line in these figures. Table 8 shows the lowest, midpoint, and maximum sample frequencies for limit disturbance according to CISPR 36.

**Table 8:** Limit of disturbance at 3 m antenna distance based on CISPR 36.

Frequency (MHz)	H Field dB (μA/m)
0.15 to 4	26.11 – 15.64 × log_10_(f_MHz_)
4 to 15	33.17 – 27.35 × log_10_(f_MHz_)
15 to 30	16.63 – 13.29 × log_10_(f_MHz_)

**Fig. 10:**
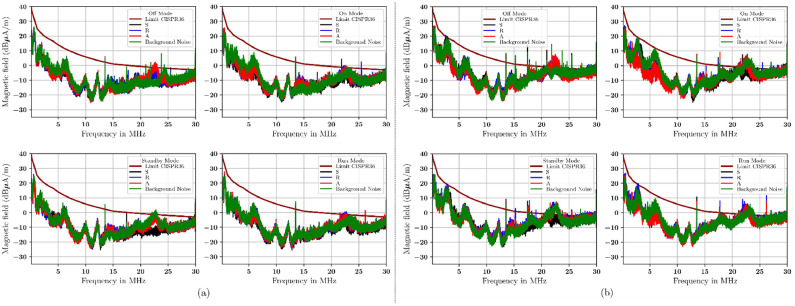
Measurement with (a) radial orientation, (b) transversal orientation at 3 m using single-layer material.

**Fig. 11:**
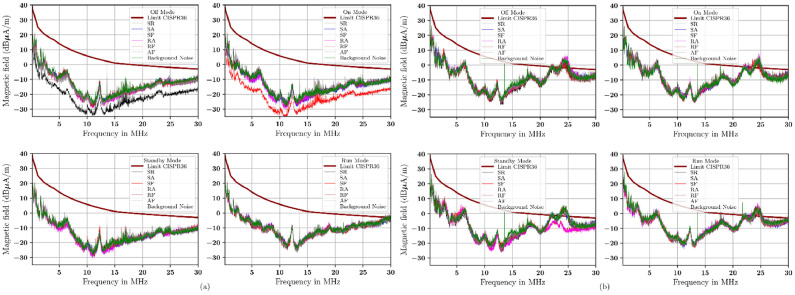
Measurement with (a) radial orientation, (b) transversal orientation at a 1 m using double-layer material.

**Fig. 12:**
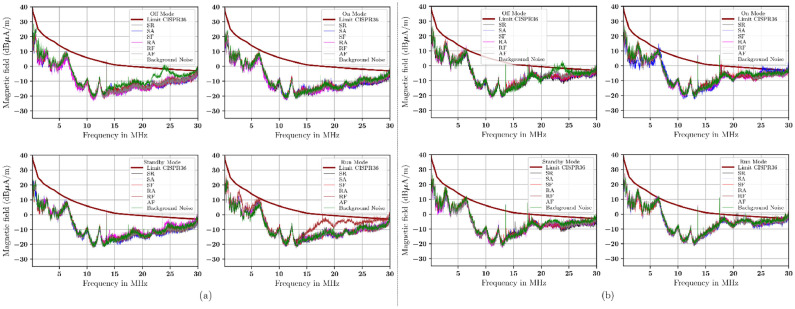
Measurement with (a) radial orientation, (b) transversal orientation at a 3 m using double-layer material.

The emission from the radial and transverse ntennas at 1 m with a single-layer material is shown in Fig. 9. For radial- and transversal-oriented antennas with single-layer materials at 3 m, see Fig. 10. The emission from the radial and transversely oriented antenna at a 1 m distance with a double-layer material is shown in Fig. 11. The emission from the radial and transverse antenna at a 3 m distance with a double-layer material is shown in Fig. 12.

In summary, the measurement show that antennas oriented transversely are higher than those oriented radially, at both 1 m and 3 m distances. The measurement results in these figures are referenced as the CISPR 36 standard. This standard helps serve as a point of reference for the measurement derived from the additional material. The signal measurement is quite high. It approaches or even exceeds the CISPR 36 limit, especially between 20 MHz and 30 MHz. This condition occurs with single-layer shielding and in combination with the materials used in this study. If we compare the measurements as seen in Fig. 9 to Fig. 10 (using a single-layer material) with those in Fig. 11 to Fig. 12 (double-layer material), then the use of a combination of materials or double-layer materials shows a significant effect and can decrease the measured signal level. The measurement shows that this double-layer material indicates a reduction of existing emissions. But in the 20 MHz to 30 MHz, not all emitted signals from the DUT can be reduced. Based on these measurements, the capability of these materials to absorb and reflect the incoming signals of each material and its combination will be calculated in percentage according to Eqs. (6) and (7) (see Table 9).

**Table 9:** The signal transmission coefficients of the rubber-based materials (in %) represent the capability of the rubber-based materials as an absorber (italic font)/reflector (bold font) in four modes of DUT operating conditions based on Eqs. (6) and (7).

Mode	Off				On				Standby				Run			
Distance	1 m	1 m	3 m	3 m	1 m	1 m	3 m	3 m	1 m	1 m	3 m	3 m	1 m	1 m	3 m	3 m
Orientation	Rad	Tran	Rad	Tran	Rad	Tran	Rad	Tran	Rad	Tran	Rad	Tran	Rad	Tran	Rad	Tran
Material	Frequency range of 0*.*15 MHz to 10 MHz															
F	*27.18*	*11.63*	**82.61**	*23.39*	*14.82*	*6.34*	**83.31**	**79.96**	**68.62**	**60.19**	**88.53**	**98.91**	**92.04**	**71.38**	**78.58**	*13.13*
R	**90.76**	*27.25*	**64.37**	**75.53**	**66.35**	*13.20*	**65.69**	*29.42*	**52.60**	*7.96*	*40.22*	**94.03**	**60.02**	**74.79**	**53.67**	**75.30**
A	**94.94**	*33.55*	*27.21*	**89.95**	**99.56**	*14.56*	*44.30*	*45.31*	*49.17*	*20.70*	**57.07**	*11.60*	**64.11**	22.92	**53.30**	**95.24**
S	**65.17**	*36.88*	*0.39*	**63.97**	*43.40*	*15.65*	*1.58*	**86.96**	**71.07**	*33.51*	*4.72*	**63.03**	**69.93**	**72.15**	**70.58**	**51.85**
SR	*0.99*	*48.07*	*5.91*	*40.32*	*5.35*	**80.93**	*16.25*	*20.16*	*44.88*	*42.69*	**75.62**	*20.67*	*36.24*	**62.15**	*43.43*	*41.07*
SA	*23.02*	**53.01**	*6.03*	**92.89**	*48.31*	**50.77**	*20.66*	*26.55*	**58.99**	**63.14**	*44.12*	*13.09*	**80.12**	*29.46*	**64.38**	*27.39*
SF	*12.85*	**68.69**	*11.55*	**70.61**	*1.06*	*21.35*	**53.08**	*47.42*	*49.64*	*5.42*	**54.03**	**69.27**	*42.72*	*34.56*	*27.12*	*28.21*
RA	*14.33*	**96.49**	*8.62*	**64.73**	*27.80*	**74.34**	*17.49*	*22.79*	*27.76*	**91.51**	*3.28*	*10.38*	**80.20**	*27.01*	*29.32*	**99.55**
RF	*24.35*	*33.03*	*24.05*	*28.17*	**70.38**	*38.03*	**51.65**	*34.73*	**93.20**	*40.19*	**55.84**	*31.52*	*35.79*	*40.44*	**96.67**	**56.87**
AF	*6.32*	**56.91**	*23.71*	*21.19*	*28.60*	**55.36**	*34.98*	*42.65*	**88.11**	**62.13**	*49.94*	*15.11*	*49.31*	*49.38*	*40.22*	*49.58*
Material	Frequency range of 10 MHz to 20MHz															
F	*27.91*	*29.40*	**62.95**	**92.29**	**84.94**	**89.17**	**61.04**	**62.42**	**55.73**	44.67	**82.89**	**95.96**	**89.10**	*38.32*	*5.83*	48.47
R	**73.47**	*49.93*	*37.71*	**67.03**	**96.02**	**66.08**	*29.56*	**60.76**	*42.74*	**90.57**	**69.27**	*12.08*	**99.37**	**84.24**	**57.41**	**91.62**
A	**85.75**	**68.49**	*77.17*	**84.74**	**89.52**	**70.87**	**94.50**	**68.14**	**66.36**	**83.24**	**65.17**	*40.88*	**51.39**	*1.41*	**84.69**	**98.34**
S	**78.92**	**98.55**	*0.65*	**71.68**	*38.23*	**68.65**	**99.17**	*12.62*	**95.91**	*28.02*	*33.47*	**68.92**	*43.27*	*35.18*	**83.44**	*28.18*
SR	*1.36*	*10.09*	*8.77*	**61.56**	*13.86*	**65.24**	**50.02**	*45.38*	*38.87*	*7.14*	**90.10**	**88.05**	**55.72**	**56.41**	**83.21**	**50.54**
SA	*40.80*	*11.71*	*8.08*	*36.77*	*6.20*	**54.61**	**79.37**	*37.62*	*25.06*	*0.70*	*45.49*	*43.32*	**77.47**	**60.47**	**65.73**	*30.05*
SF	*28.45*	*0.11*	*6.80*	**64.17**	*5.23*	**62.89**	**66.66**	*25.18*	**83.29**	*2.89*	**95.68**	*11.36*	*38.08*	**59.66**	**63.23**	*47.64*
RA	*8.84*	*17.50*	*2.53*	*18.52*	*3.11*	**84.81**	*40.48*	**56.02**	*9.78*	*15.17*	**56.33**	*45.83*	**56.70**	**95.96**	**82.41**	**88.89**
RF	*42.76*	*15.49*	*8.93*	**55.49**	*35.56*	*48.49*	**86.44**	*30.94*	**50.73**	*0.40*	**99.58**	**95.21**	*30.28*	*31.36*	**97.41**	**96.70**
AF	*19.34*	*15.08*	*5.24*	*8.47*	**90.09**	**61.62**	**51.87**	**79.05**	**74.38**	*11.51*	*47.34*	*37.48*	*24.44*	**52.65**	**73.36**	*34.60*
Material	Frequency range of 20 MHz to 30MHz															
F	**74.33**	**60.06**	**86.93**	**93.96**	*48.91*	**94.51**	**65.99**	**76.05**	**83.25**	*27.81*	**89.13**	**95.61**	**61.52**	**82.40**	*2.25*	*46.55*
R	**89.17**	*36.53*	*46.39*	*21.85*	*47.09*	*20.20*	*46.70*	**61.58**	*43.50*	**85.62**	**88.28**	*5.96*	**56.23**	**92.36**	*47.20*	**92.31**
A	**88.58**	**50.38**	**80.18**	**82.86**	**73.58**	*24.06*	**89.81**	**58.32**	*31.29*	**54.37**	*0.96*	*18.96*	**70.71**	*49.76*	**89.07**	**98.32**
S	**94.55**	41.38	**67.60**	**80.07**	*36.16*	**63.56**	**98.50**	*4.30*	**93.17**	*17.09*	*30.03*	**61.07**	**61.41**	*44.79*	**87.90**	*22.11*
SR	*16.87*	*29.66*	*0.44*	*6.51*	*33.27*	*32.35*	*8.79*	*19.86*	**60.81**	*39.90*	*14.54*	*3.50*	**60.39**	**70.77**	**70.75**	*25.48*
SA	*5.55*	*3.15*	*0.19*	*9.18*	*14.20*	*44.03*	*15.85*	**55.86**	*5.57*	*24.59*	*11.63*	*13.30*	**91.18**	**74.92**	**67.24**	*18.55*
SF	*16.77*	*10.86*	*0.10*	*10.97*	*25.51*	**72.36**	*32.50*	*24.59*	*36.07*	**76.39**	*22.10*	*26.30*	*36.95*	**72.14**	**60.95**	*39.24*
RA	*30.41*	*22.79*	*0.47*	*12.80*	*48.07*	**80.15**	*31.18*	*18.49*	*38.29*	**55.13**	**78.86**	16.13	**65.95**	**87.84**	**76.61**	**51.84**
RF	*35.42*	*27.36*	*0.75*	*14.30*	**66.57**	*41.00*	*37.58*	*18.83*	*41.46*	*30.59*	*20.17*	*8.51*	*26.91*	*40.70*	**99.82**	**78.65**
AF	*13.30*	*9.79*	*0.49*	*32.88*	**54.14**	*14.28*	*43.71*	*36.92*	**52.65**	*13.73*	*23.87*	*20.19*	*33.68*	**68.79**	**60.51**	*31.11*

Note 1 - For Single-layer material: F = conductive fabric, R = rubber, A = antistatic mat, and S = silicon.

Note 2 - For Double-layer material: SR = silicon/rubber, SA = silicon/antistatic mat, SF = silicon/conductive fabric, RA = rubber/antistatic mat, RF = rubber/conductive fabric, AF = antistatic mat/conductive fabric.

Table 9 presents the signal transmission coefficients of the rubber-based materials. The table shows the average signal measurements taken at distances of 1 m and 3 m. It includes both radial and transverse antenna orientations. To clarity, "1 m Rad" means measurements at 1 m using a radial antenna. "1 m Tran" refers to measurements at 1 m with a transverse antenna, and so forth. Italic fonts show the materials’ absorption coefficients. These coefficients indicate the percentage of the signal absorbed by the electronic module parts in the electric vehicles. Bold fonts show the reflection coefficient of the material which indicates the percentage of signals that are the reflected.

A single material antistatic mat works in the frequency range of 0.15 MHz to 10 MHz. Its signal transmission coefficient is 56.25 %. In the frequency range between 10 MHz and 20 MHz, the silicon material has an absorption capability of 43.75 %. Also, across the frequency range of 20 MHz to 30 MHz, the absorption capability of all materials absorbed less than 40 %.

Table 10 shows the absorption effectiveness (in percent) of the single-layer and two-layer rubber materials. This data covers all operational modes: Off, On, Standby, and Run. The table has three frequency ranges: 0.15 MHz to 10 MHz, 10 MHz to 20 MHz, and 20 MHz to 30 MHz. It lists material combinations exceeding 50 % of the occurrences in the test results. These materials show absorption capability at the CISPR 36 frequencies. It outlines the measurement distances of 1 m and 3 m. It also notes the antenna orientations: radial and transverse. These are based on DUT’s operational mode during the measurement.

**Table 10:** Number of occurrences of rubber-based materials (in %) with absorption capability in the test result in Table 9.

Material	All Modes	%	Mode On, Standby, Run	%
	Frequency range of 0*.*15 MHz to 10MHz			
F	6	37*.*50	3	18*.*75
R	5	31*.*25	4	25*.*00
A	9	56.25	7	43*.*75
S	7	43*.*75	5	31*.*25
SR	13	81.25	9	56.25
SA	9	56.25	7	43*.*75
SF	11	68.75	9	56.25
RA	10	62.50	8	50.00
RF	10	62.50	6	37*.*50
AF	12	75.00	9	56.25
	Frequency range of 10 MHz to 20 MHz			
F	6	37*.*50	4	25*.*00
R	5	31*.*25	3	18*.*75
A	2	12*.*50	2	12*.*50
S	8	50.00	7	43*.*75
SR	7	43*.*75	4	25*.*00
SA	11	68.75	7	43*.*75
SF	9	56.25	6	37*.*50
RA	9	56.25	5	31*.*25
RF	9	56.25	6	37*.*50
AF	9	56.25	5	31*.*25
	Frequency range of 20 MHz to 30 MHz			
F	4	25*.*00	4	25*.*00
R	9	56.25	6	37*.*50
A	5	31*.*25	5	31*.*25
S	7	43*.*75	6	37*.*50
SR	12	75.00	8	50.00
SA	12	75.00	8	50.00
SF	12	75.00	8	50.00
RA	9	56.25	5	31*.*25
RF	13	81.25	9	56.25
AF	12	75.00	8	50.00

Table 10 shows the percentage capability of the single-layer and two-layer rubber-based materials used in this study to absorb signals (in %). This is mesured in percentages for all modes except Off mode. The modes included are Mode On, Standby, and Run). Only those with 50 % or more occurrences are listed. The table has three frequency ranges, namely 0.15 MHz to 10 MHz, 10 MHz to 20 MHz, and 20 MHz to 30 MHz. It shows the materials combinations for certain modes at distances of 1 m and 3 m with antennas with both radial and transverse orientation according to the DUT mode conditions. The data is presented as a percentage of the total mode combinations.

The following is a summary of the rubber-based materials signal transmission coefficients (in %). They must be 50% or higher (see Table 10).

This study recommends using rubber-based materials for absorbers in 0.15 MHz and 10 MHz range. The suggested materials are SR (56.25 %), SF (56.25 %), and AF (56.25 %). Provided the percentage criteria are reduced to 50 %, and even though radiated emissions escape from the DUT, it is possible to combine rubber with antistatic mat (RA) materials at 50 %.

In the 10 MHz to 20 MHz range, no material combination that achieves a signal transmission coefficient over 50 %. But a combination of SA and S can reach a maximum absorption of 43.75 %.

In the 20 MHz to 30 MHz range, the absorber material combination of rubber with conductive fabric (RF) is accountable for approximately 56.25 % of the absorption. The combination of SR, SA, SF, and handles roughly 50 % of the absorption. In these frequency ranges, three distinct materials can be employed to protect the electronic modules from potential emission disturbances.

The value S_21_ was measured in the chamber. The difference between value of measured S_21_ with shielded material and S_21_ without shielded material was calculated to get absorption capability. The results in Table 10 showed that the absorption capability of combining silicon and rubber ranged from 25 % and 81.25 %. The absorption capability is not uniform across all tested frequencies.

The significant emission absorption in the frequency ranges of 0.15 MHz to 10 MHz and 20 MHz to 30 MHz was primarily due to a mixture of materials comprising SR, SF, and AF. This combination accounted for 50 % and 56.25 % of the total absorption. A combination of RF materials can be used for measurements between 20 MHz and 30 MHz, if their absorption rate exceeds that of the alternative materials.

Table 11 displays the average shielding effectiveness of various types of material across difference frequency ranges. In the 0.15 to 4 MHz range, single-material rubber has a shielding effectiveness of 37.742 dB. In the frequency range of 4 – 15 MHz and 15 – 30 MHz, single-material silicon shows a shielding effectiveness of 37.492 dB and 37.567 dB, respectively. The best shielding effectiveness between 0.15 and 4 MHz comes from a combination of the materials silicon and rubber. It achieves 75.479 dB. In the 4 and 15 MHz range, the best absorption comes from a combination of silicon and fabric material. It reaches 74.942 dB. Moreover, the combination of silicon and rubber reaches 75.028 dB between 15 and 30 MHz.

**Table 11:** indicates the values of shielding effectiveness on frequency ranges of limit defined on CISPR 36 (Table 8).

Table 11. Shielding effectiveness across different frequency ranges and various types of material.Type of material	Frequency range (MHz)	SE(Avg) (dB)
Silicon (S)	0.15 – 4	37.737
	4 – 15	37.492
	15 – 30	37.567
Rubber (R)	0.15 – 4	37.742
	4 – 15	37.362
	15 – 30	37.461
Antistatic Mat (A)	0.15 – 4	37.605
	4 – 15	37.424
	15 – 30	37.444
Fabric (F)	0.15 – 4	37.661
	4 – 15	37.450
	15 – 30	37.458
S/R	0.15 – 4	75.479
	4 – 15	74.854
	15 – 30	75.028
S/A	0.15 – 4	75.342
	4 – 15	74.916
	15 – 30	75.011
S/F	0.15 – 4	75.399
	4 – 15	74.942
	15 – 30	75.025
R/A	0.15 – 4	75.347
	4 – 15	74.787
	15 – 30	74.905
R/F	0.15 – 4	75.347
	4 – 15	74.787
	15 – 30	74.905
A/F	0.15 – 4	75.266
	4 – 15	74.875
	15 – 30	74.902

According to CISPR 36, the limit of disturbances in 0.15 – 4 MHz is a maximum of 26.11 dB. Rubber shielded material offers the best shielded effectiveness in this frequency range. It is recommended to be used as the sole shielding material to reduce emission sources from EVs. In addition, a combination of silicon and rubber can reduce emission of EV by 74.942 dB. In the frequency range of 4 – 15 MHz, silicon as a single shielding material and combined with fabric can be used as shielding to reduce EMI. In the third frequency range of CISPR 36, silicon as a single material and combined with rubber is recommended as shielding.

In addition, the measurement uncertainties are calculated from measurement apparatus used. Uncertainty in antenna loop factor obtained from certificate of calibration is 0.87 dB. The uncertainty of signal hound receiver obtained from datasheet is 2 dB. The uncertainty of measuring cable is calculated as 0.07 dB. The combined standard uncertainty of this measurement is 2.29 dB with expanded uncertainty of 4.57 dB.

## Limitations

This method proposes the use of relatively affordable prices of rubber-based materials as absorbers. The method can reduce EMI within the CISPR 36 standard limits, especially at low-frequency. This study investigates alternative novel EMI mitigation methods within this frequency range. It uses organic materials as absorbers, which can be applied to reduce the potential emission disturbances from or to electronic modules of the electric vehicles.

The device under test (DUT) focused on electric vehicles, mainly EVs for towing goods. The proposed absorber material in this manuscript was limited to rubber-based materials. These materials cover electronic modules on this DUT. The efficacy of the shielded material relies on the manufacturer, even if the materials are similar. The effectiveness of EMI suppression was evaluated separately, but it was only in combination of these rubber-based materials.

The method involved applying the shielded material directly to the EV parts, like the battery, inverter, and other potential sources of EMI. The scalability can be achieved by mounting the shielded material directly into EV parts. This method can help reduce the potential emission during the fabrication process.

## Ethics statements

This study does not involve any human and animal subjects; during the measurement period, only measurement apparatus were used for measuring the EM emission from the EV as a DUT in an open-area test site and for measuring shielding materials inside a semi-anechoic chamber.

Moreover, this study does not collect any data from social media platforms, and references obtained from internet sources are mentioned in the references section.

## Related research article

None.

## For a published article

None.

## CRediT author statement

**Arief Rufiyanto**: Writing original draft preparation, review & editing, Methodology, Investigation, Formal analysis, Data curation, Conceptualization, Validation. **Gamantyo Hendrantoro**: Writing review, Supervision, Methodology, Conceptualization. **Reza Septiawan**: Writing review & editing, Methodology, Supervision, Conceptualization, Formal analysis, Data curation. **Eko Setijadi**: Methodology, Supervision, Validation, Conceptualization. **Budi Sulistya**: Visualization, Resources. **Sardjono Trihatmo**: Validation, Resources.

## Declaration of competing interest

The authors declare that they have no known competing financial interests or personal relationships that could have appeared to influence the work reported in this paper.

## Data Availability

Data will be made available on request.
